# Partial Reactions of the Na,K-ATPase: Determination of Activation Energies and an Approach to Mechanism

**DOI:** 10.1007/s00232-020-00153-y

**Published:** 2020-11-13

**Authors:** Hans-Jürgen Apell, Milena Roudna

**Affiliations:** Department of Biology, University of Konstanz, 78464 Konstanz, Germany

**Keywords:** Sodium pump, Active ion transport, Post-Albers cycle, Reaction kinetics, Fluorescence, Molecular mechanism

## Abstract

**Abstract:**

Kinetic experiments were performed with preparations of kidney Na,K-ATPase in isolated membrane fragments or reconstituted in vesicles to obtain information of the activation energies under turnover conditions and for selected partial reactions of the Post-Albers pump cycle. The ion transport activities were detected with potential or conformation sensitive fluorescent dyes in steady-state or time-resolved experiments. The activation energies were derived from Arrhenius plots of measurements in the temperature range between 5 °C and 37 °C. The results were used to elaborate indications of the respective underlying rate-limiting reaction steps and allowed conclusions to be drawn about possible molecular reaction mechanisms. The observed consequent alteration between ligand-induced reaction and conformational relaxation steps when the Na,K-ATPase performs the pump cycle, together with constraints set by thermodynamic principles, provided restrictions which have to be met when mechanistic models are proposed. A model meeting such requirements is presented for discussion.

**Graphic Abstract:**

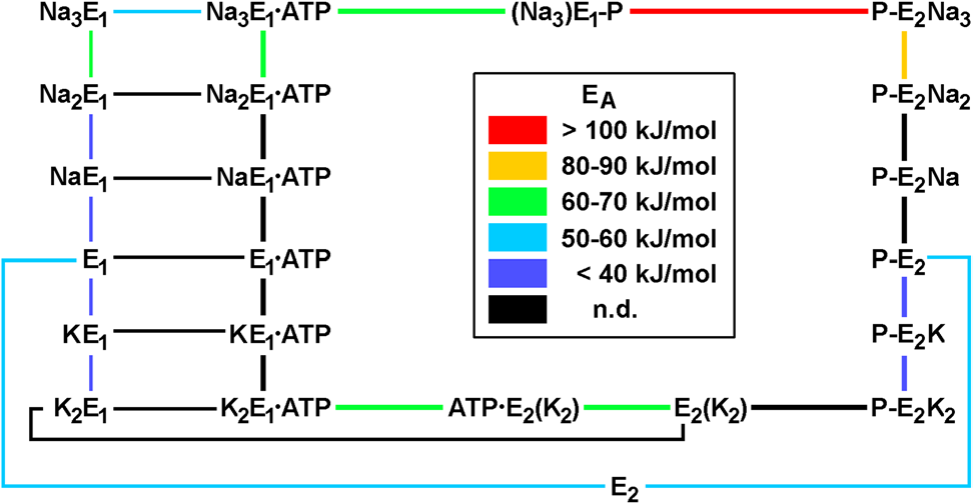

## Introduction

In most animal cells electrochemical potential gradients across the cytoplasmic membrane are generated directly or indirectly by the Na,K-ATPase. From a thermodynamical point of view, this protein can be understood as a molecular machine that converts chemical energy, stored in the compound ATP, to an electrochemical potential gradient across the cell membrane by pumping three sodium ions out of the cytoplasm and two potassium ions in opposite direction per each turnover. Numerous publications and reviews are available presenting information on structural aspects (Morth et al. 2007; Shinoda et al. 2009; Kanai et al. 2013; Nyblom et al. 2013), enzymatic and transport properties(Glynn 1985; Kaplan 1985; Jørgensen et al. 2003; Apell 2019), electrogenicity (De Weer et al. 1988; Apell 2004; Gadsby 2009), and energetics (Läuger 1984, 1991).

**Fig. 1:**
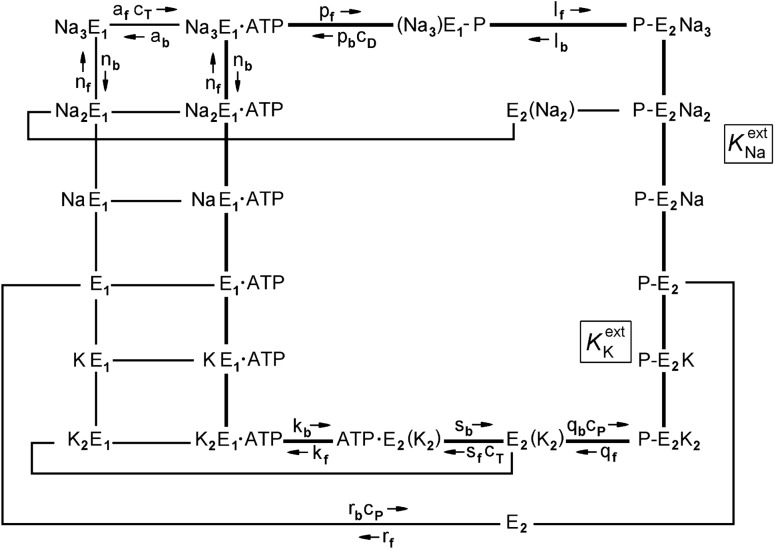
Extended Post-Albers pump cycle of the Na,K-ATPase. Bold lines represent the physiological pump mode. Reaction steps labeled by rate constants were accessible in kinetical experiments, analyzed, and used in numerical simulations according to model based on this reaction cycle (Heyse et al. 1994). *K*_Na_^ext^ and *K*_K_^ext^ are the apparent equilibrium dissociation constants of Na^+^ and K^+^ binding, respectively, from the external side of the membrane. (Na_3_)E_1_-P, E_2_(K_2_), and ATP‧E_2_(K_2_) are occluded states in which the bound ions are unable to exchange with either aqueous phases of membrane

In its normal mode of operation, the enzyme performs a pump cycle composed of conformational transitions as well as ligand binding and release steps. Spectroscopic and proteolytic studies indicated that the enzyme can assume two principal conformations, designated as E_1_ and E_2_. The E_1_ conformation is stabilized by Na^+^ and characterized by ion-binding sites facing the cytoplasm; the E_2_ conformation is stabilized by K^+^ and the ion-binding sites are accessible from the extracellular medium in the phosphorylated state of E_2_. From enzymatic and transport studies under various conditions, a reaction cycle was proposed (Albers 1967; Post et al. 1972) that is shown in an extended form in Fig. 1. The bold lines represent the physiological mode of ion pumping, the so-called Post-Albers cycle. The other pathways account for the so-called non-canonical pump modes (Glynn 1985; Apell 2019).

When under physiological conditions, the protein is phosphorylated by ATP in the E_1_ conformation, the bound Na^+^ becomes “occluded”, i.e., trapped inside the protein (Na_3_E_1_ATP → (Na_3_)E_1_-P) (Glynn et al. 1984). After transition to the P-E_2_ conformation, Na^+^ is released and exchanged against two K^+^. The resulting occupation of the ion-binding sites by K^+^ leads to dephosphorylation of the protein and simultaneous occlusion of K^+^. The pump cycle is completed by transition back to the E_1_ conformation and subsequent exchange of 2 K^+^ against 3 Na^+^ on the cytoplasmic side. When the addition of a substrate triggers a limited number of reaction steps, such a sequence is named partial reaction.

To gain insights into the molecular mechanism that controls the changes in the protein when a single reaction step of the pump cycle or more complex partial reactions take place, an empirical quantity named activation energy is an interesting indicator to discriminate at least between kinds of processes. Reactions that exhibit activation energies larger than 50 kJ/mol (or Q_10_ > 2) involve considerable entropy effects that have to be attributed to structural or conformational rearrangements (Gutfreund 1995). Smaller activation energies down to the order of 20 kJ/mol may be assigned to ligand coordination in binding sites without significant conformational rearrangements, and still lower values may be assumed to represent diffusion-controlled processes.

To determine the activation energy of single step in a complex reaction cycle like the Post-Albers cycle is not an easy undertaking both experimentally and theoretically. Therefore, for a long time, only the overall activation energies of ion pumps working under steady-state conditions were investigated (Post et al. 1965; White and Blostein 1982; Marcus et al. 1986; Esmann and Skou 1988). There are, however, at least two considerable reasons to study the activation energies of single reaction steps in the transport cycle: (1) In various laboratories experiments to study specific partial reactions were performed at different temperatures. To compare the results, data have to be adjusted to the same temperature. To do so, for the most part, an average activation energy of 55 kJ/mol was assumed. This has to be justified or adjusted. (2) When the activation energy of a transition between states of the ion pump is known (i.e. of the forward and backward rate or of the equilibrium constant), one can deduce the enthalpy and entropy terms of the reaction by application of basic thermodynamic principles. This is important information on the energetics of the enzyme and provides at least hints on the underlying molecular mechanism taking place in the monitored reaction step(s).

Establishing activation energies under steady-state conditions of ATP-hydrolyzing activities (Esmann and Skou 1988) or of ion-pump rates (Apell et al. 1985, 1990) is possible by straight forward approaches. An analysis of partial reactions of the pump cycle requires, however, that this reaction consists of only a single step or that at least the activation energy of the rate-limiting step is well separated from that of the others steps in this sequence. Nonlinear Arrhenius plots are an indication that different steps are rate-limiting at the low- and high-temperature range of the experiment (Gutfreund 1995). Phase transitions in the lipid phase may be excluded as cause since the transition temperatures of the prominent lipid species are below the temperature range covered by the experiments, and phase transitions occur in narrow temperature windows compared to the transition ranges of > 10 °C observed here.

In the following, experiments are presented which allowed the determination of the activation energies of a series of rate and binding constants and fits of experiments by a mathematical description based on the reaction cycle shown in Fig. 1.

On the occasion of Dr. Robert L. Post’s hundredth birthday we dedicate this paper to him, a pioneer who paved the way for numerous scientists in the field of understanding the function of the Na,K-ATPase.

## Materials and Methods

### Materials

Dioleoyl phosphatidyl choline was purchased from Avanti Polar Lipids, Alabaster, Alabama. Phosphoenolpyruvate, pyruvate kinase, lactate dehydrogenase, NADH, and ATP (disodium salt, special quality) were supplied from Roche Life Science, and apyrase VI and ouabain from Sigma-Aldrich. Oxonol VI, 5-iodoacetamidofluorescein (5-IAF), RH237, RH421, and P^3^-1-(2-nitro)phenylethyladenosine-5′-triphosphate (caged ATP) were purchased from Molecular Probes (Eugene, Oregon). Na^+^ and K^+^ salts were used in Suprapur quality (Merck). All other reagents were obtained at the highest quality available.

### Enzyme Preparation

Purified Na,K-ATPase was prepared from the outer medulla of rabbit kidneys using a slightly modified procedure C of P.L. Jørgensen (Jørgensen 1974b; Apell et al. 1985). This method yields enzyme in the form of open membrane fragments containing about 0.8 mg phospholipids and 0.2 mg cholesterol per mg protein (Jørgensen 1974a). Enzyme activity was determined by the pyruvate kinase/lactate dehydrogenase assay (Schwartz et al. 1971). The specific Na,K-ATPase activity of the different preparations used was found in the range between 1900 and 2400 μmol P_i_ per hour and mg protein at 37 °C. To obtain the activation energy of the ATPase according to the theory of S. Arrhenius, ATP-hydrolyzing activity of the enzyme had to be measured at different temperatures. Typically, experiments were performed at temperatures between 8 °C and 37 °C. In this temperature range, the reaction of the detection assay was fast compared to the enzyme activity in the solution so that the determined activation energies were not distorted by the method.

### Vesicle Preparations

Reconstituted vesicles with incorporated Na,K-ATPase were prepared as described previously (Apell et al. 1985). In short, Na,K-ATPase was solubilized in sodium cholate from purified open membrane fragments. After addition of dioleoylphosphatidyl choline dissolved in sodium cholate, the detergent was removed by dialysis at 4 °C for at least 60 h. This procedure yielded unilamellar vesicles with a diameter of about 90 nm (Marcus et al. 1986). The dialysis buffer contained Na_2_SO_4_ and K_2_SO_4_ in various concentrations with a constant total of 75 mM SO_4_^2−^, and 5 mM MgSO_4_, 30 mM imidazole, and 5 mM EDTA. Sulfate was chosen as anion to minimize leak conductance. Buffer pH was adjusted at 7.2.

### Fluorescence Labeling

Fluorescence labeling of the enzyme with 5-IAF was performed by incubating 200–300 μg of enzyme for 48 h at 4 °C with a solution containing 100 μM 5-IAF, 10 mM K_2_SO_4_, and 50 mM imidazole sulfate and pH 7.5 (Kapakos and Steinberg 1982; Stürmer et al. 1989). The labeled enzyme was separated from unbound dye by passing the reaction mixture through a short Sephadex G-25 column.

### Fluorescence Measurements

Steady-state fluorescence measurements were carried out with a Perkin-Elmer LS 50B luminescence spectrometer. The thermostated cell holder was equipped with a magnetic stirrer. In the experiments with oxonol VI, RH421, and RH237, the dye was added from an ethanolic stock solution to the buffer solution before addition of membrane fragments or vesicles.

Oxonol VI was used at a final concentration of 30 nM to detect the membrane potential across the vesicle membrane with an excitation wavelength of 580 mm (slit width 20 nm) and the emission wavelength of 660 nm (slit width 5 or 10 nm) (Apell and Bersch 1987). For experiments with RH421 or RH237, the excitation wavelength was set to 580 nm (slit width 15 nm) and the emission wavelength to 660 nm (slit width 20 nm) as published before (Stürmer et al. 1991; Bühler et al. 1991; Schneeberger and Apell 1999). The electrochromic styryl dyes were used to detect electrogenic partial reactions of the Na,K-ATPase. Typical dye concentrations were 200 nM.

When the electrogenic pump activity of Na,K-ATPase reconstituted in lipid vesicles was measured with oxonol VI, the most significant information from these experiments was the initial slope of the membrane potential increase after activation of the pumps by ATP (Apell and Bersch 1988). This slope is proportional to the pump rate of the Na,K-ATPase.

To trace the course of fluorescence, signals were measured with high time resolution (at a frequency of 1000 Hz), triggered by photolytic release of ATP from caged ATP with a specifically devised home-made setup (Stürmer et al. 1989). A cylindrical quartz cuvette was filled with 200–300 µl of a suspension of membrane fragments (usually 5–30 µg protein per ml) in a medium containing 30 mM imidazole buffer, pH 7.2, 1 mM EDTA, 5 mM MgCl_2_, and 150 mM NaCl. The fluorescence was excited by light from a 250 W tungsten-halogen lamp in combination with a narrow-band interference filter of 590 nm (half-width about 10 nm) or by a HeNe Laser emission at 594 nm. Fluorescence light emitted from the sample cell was collected by an ellipsoidal mirror and focused onto the cathode of a photomultiplier. The emission wavelength was selected by interference filters of 660 nm (half-width about 13 nm) and an UV-cutoff filter (< 350 nm). ATP was released with a time constant of 4.6 ms (pH 7.0) from its inactive precursor, caged ATP (McCray et al. 1980), in the cuvette by a UV-light flash (wavelength 308 nm, total energy 150 mJ, duration 10 ns) generated with a EMG 100 excimer laser (Lambda Physics, Göttingen). A photochemical yield of 15 to 25% was determined. In order to remove traces of free ATP in the sample of caged ATP, a small amount of apyrase VI (10^–3^ units/ml) and 2 mM Mg^2+^ were added to the membrane suspension prior to the flash experiment.

### Numerical Analysis

To describe the temperature dependence of pump activity, the rate constants and equilibrium dissociation constants have to be defined as temperature-dependent processes according to the Arrhenius equation

1 $$k_{i} (T) = A_{i} \cdot \exp \left( {\frac{{ - (E_{A} )_{i} }}{RT}} \right)$$

where *k*_i_ is the rate constant, *A*_i_ is a temperature-independent factor, (*E*_A_)_i_ is the activation energy of the rate constant *k*_i_, *R* is the gas constant, and *T* the absolute temperature. *A*_i_ can be replaced by a known value of *k*_i_ at *T* = 20 °C, which modifies Eq. 1 in the following way

2 $$\tt \tt k_{i} (T) = k_{i} \left( {293\,K} \right) \cdot \exp \left( {\frac{{ - (E_{A} )_{i} }}{R} \cdot \left( {\frac{1}{T} - \frac{1}{293\,K}} \right)} \right)$$

The activation energies (*E*_A_)_i_ for all the different rate constants of the different reaction steps were obtained by fit of a regression line to data sets obtained at various temperatures. The same relation holds also for the equilibrium dissociation constants, *K*_i_(*T*).

## Results

### Activation Energy of the Na,K-ATPase Under Turnover Conditions

The temperature dependence of the ATP-hydrolyzing and ion-pumping activity of the Na,K-ATPase was measured under steady-state conditions. The enzyme was investigated in purified membrane fragments or reconstituted in vesicles. When studying pump activity in vesicles, reconstitution of the Na,K-ATPase was performed in buffers containing 140 mM K^+^  + 10 mM Na^+^ or 150 mM Na^+^, while the measuring buffer contained either 140 mM Na^+^  + 10 mM K^+^ or 150 mM Na^+^, always with sulfate as anion. When the Na,K-ATPase was reconstituted in vesicles, the activity of the pump could be measured either enzymatically by its ATP-hydrolyzing activity or optically by the detection of the increasing transmembrane electrical potential using oxonol VI. ATP-hydrolyzing activity could be obtained also from experiments with membrane fragments. Both methods detect steady-state activities after addition of ATP to start pump turnover.

#### a) ATP-Hydrolyzing Activity

**Fig. 2:**
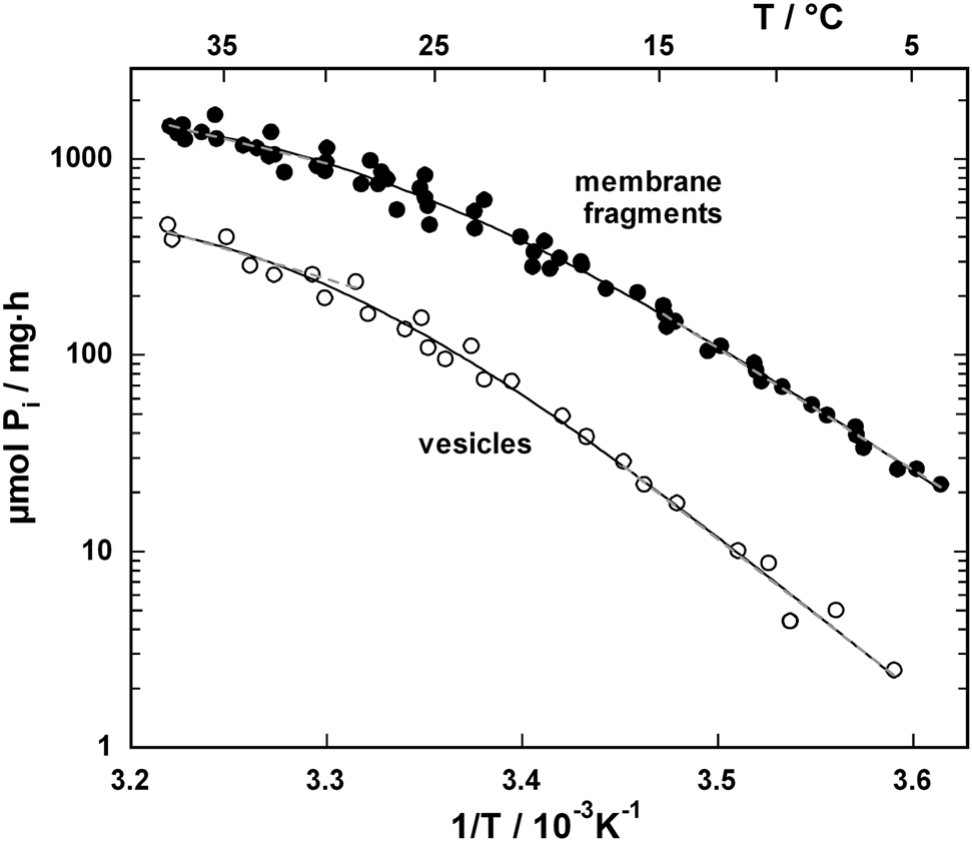
Arrhenius plot of the ATP-hydrolyzing activity of the Na,K-ATPase in purified open membrane fragments from the outer medulla on rabbit kidney (“membrane fragments”) and after reconstitution of the isolated Na,K-ATPase in lipid vesicles (“vesicles”). The line through the data points was drawn to guide the eyes. The bent form indicates that at low and high temperatures different reaction steps are rate limiting. In the temperature range between 5 °C and 15 °C as well as 30 °C and 37 °C linear regression lines were fitted through the data (dashed lines) to determine the activation energies from the slope of these lines

According to Schwartz and coworkers (Schwartz et al. 1971), the ATP-hydrolyzing activity can be spectroscopically determined by an assay in which the produced ADP is rephosphorylated stoichiometrically by soluble enzymes consuming NADH. To exclude any distorting effect of the enzyme assay on the detected rate of ATP hydrolysis, all experiments were performed with one and fivefold concentration of enzyme or vesicles. It was verified that the rate of NADH consumption was linearly proportional to the Na,K-ATPase concentration. Results of the ATPase activity from both kinds of enzyme preparations are shown in Fig. 2 in the form of an Arrhenius diagram. In experiments with reconstituted vesicles, it was checked that the presence or absence of 100 nM oxonol VI caused no difference in ATP-hydrolyzing activity. This observation indicated that there is no interference between protein function and the fluorescent dye at the concentration of 30 nM as used in the experiments.

The enzyme activity was determined in the range between 5 and 37 °C and a bent temperature dependence was found in all series of experiments. The Arrhenius plots were linear at the low- and high-temperature range of the collected data, i.e., between 5 °C and 15 °C as well as between 30 °C and 37 °C. From the slopes activation energies *E*_A_ of 118  ±  2.5 kJ/mol at lower and 45.9  ±  8.7 kJ/mol higher temperatures were determined, respectively, for the membrane fragments, as well as 147.5  ±  13 kJ/mol and 56.6  ±  11 kJ/mol in the case of enzyme reconstituted in vesicles. The significantly decreased activation energies at high temperatures indicated a change in the rate-limiting step of the reaction cycle. In Fig. 2 data of two or three independent series of experiments are merged. The hydrolyzing activity of the reconstituted enzyme was calculated under the assumption of the average of 0.8 g protein/μl vesicle. The reduced specific enzyme activity derived from experiments with vesicle preparations was caused by the fact that only about half the ion pumps were reconstituted with an “inside-out” and by a different (suboptimal) lipid composition of the reconstituted vesicle membrane (Marcus et al. 1986). The latter argument is supported also by the increased activation energies observed in case of the reconstituted Na,K-ATPase (Marcus et al. 1986).

#### b) Electrogenic Ion Transport

Potential sensitive dyes were used to detect the electrogenic activity of reconstituted transport proteins. It was shown (Apell et al. 1990) that the most significant parameter, which can be obtained from these experiments with the Na,K-ATPase, is the initial slope of the fluorescence increase after starting the pump activity by addition of ATP. It was derived that the slope is proportional to the turnover rate of the pump (Apell and Bersch 1988). During this initial phase neither membrane potential nor intravesicular K^+^ concentration changed significantly, therefore, the pump rate of the enzymes was not affected notably.

**Fig. 3:**
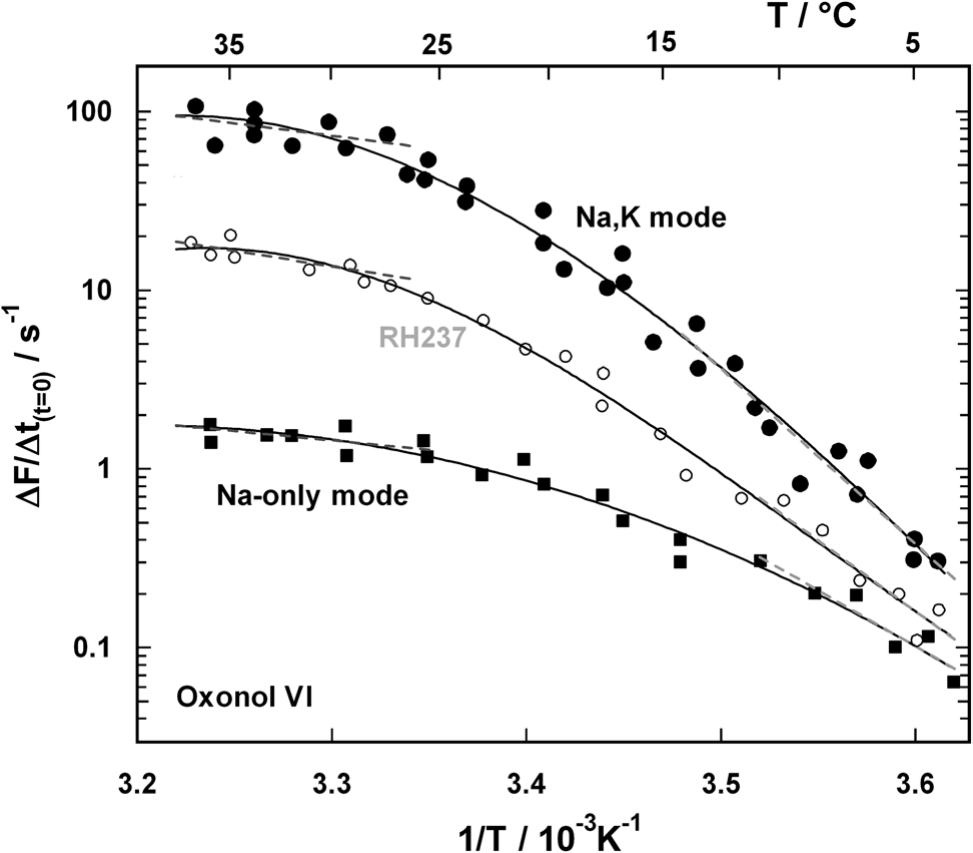
Arrhenius plot of the electrogenic pump activity of the Na,K-ATPase. The pump rate is proportional to the fluorescence increase, Δ*F*/Δ*t*_(t=0)_ upon addition of ATP at *t* = 0. The ion pumps were reconstituted in lipid vesicles and measured in the Na,K mode with two fluorescent dyes (oxonol VI and RH237) and in the Na-only mode with oxonol VI. The fluorescence increase is proportional to the increase of the membrane voltage.(Apell and Bersch 1988) The bent course of the data let to different activation energies at low- and high-temperatures which were calculated at low temperatures and estimated at high temperatures from regression lines through the data (dashed lines)

The initial slope of the normalized fluorescence increase, ∆*F*_norm_ = (*F*—*F*_0_)/*F*_0_, with *F*_0_ = *F*(*t* = *0*), was measured as a function of temperature. Two different potential sensitive dyes were used (oxonol VI and RH237). To study the Na,K mode of the pump; in different series of experiments the extravesicular (= cytoplasmic) buffer contained 140 or 148 mM Na^+^ and 10 or 2 mM K^+^, respectively. In these experiments, no difference of the initial slope was found for both K^+^ concentrations. The vesicles were filled with 140 mM K^+^ and 10 mM Na^+^. The Arrhenius plots were linear in the temperature range between 5 °C and 15 °C in case of both dyes with the same activation energies, which were calculated from regression lines though the data to be *E*_A_ = 174  ±  15 kJ/mol (oxonol VI) and *E*_A_ = 172  ±  24 kJ/mol (RH237) (dashed lines in Fig. 3). The smaller initial slope of the fluorescence signal obtained in the case of RH237 has to be attributed to the significantly lower specific fluorescence changes of this dye. In the studies of both fluorescence dyes, the temperature dependence of the initial slope, ∆*F/*∆*t*, was bent to sublinear behavior above 20 °C, supporting the findings observed in the ATP-hydrolyzing experiments. At high temperatures, the activation energy of the rate-limiting step could only be approximated to be about 32 kJ/mol in both sets of experiments (dashed lines in Fig. 3). An additional series of experiments was performed to determine the temperature dependence of the initial pump rate in the Na-only mode with oxonol VI as potential-sensitive dye. This pump mode was provoked by performing experiments without K^+^ in the buffer inside and out of the vesicles. The bend in the temperature dependence was similar to that of the Na,K-mode, but the activation energy was 120  ±  20 kJ/mol in the linear range between 5 °C and 20 °C (Fig. 3). In the temperature range above 25 °C an activation energy of ~ 15 kJ/mol was estimated. At 20 °C the pump rate derived from the initial slope was about 25 times higher in the Na,K mode when compared to the Na-only mode.

### Activation Energies of Single Steps in the Pump Cycle

The temperature dependence of the overall pump rate is a property in which the temperature dependence of all participating reactions steps is superimposed in a complex manner. It has to be expected that rate-limiting steps in the pump cycle contribute most significantly to the turnover rate. Without further experimental approach, however, the role of the various partial reactions may not be specified. Therefore, it is important to ascertain conditions by which single partial reactions can be isolated so that it becomes possible to study them individually. In the following, a number of the experimentally accessible steps are identified and analyzed.

To obtain information on single reaction steps of the Na,K-ATPase, two different experimental approaches were used. In one set of experiments, the protein in membrane fragments was labeled with 5-IAF, which allowed the detection of the conformation transitions between the states of E_1_, P-E_2_, and E_2_(K_2_)/P-E_2_(K_2_) by changes of the fluorescence amplitude (Stürmer et al. 1989). Another set of experiments utilized the properties of the styryl dye RH421, which modifies its fluorescence emission when ions bind or are released between different states of the enzyme. These experimental techniques are extensively discussed elsewhere (Bühler et al. 1991; Stürmer et al. 1991; Schneeberger and Apell 1999).

### Binding of Sodium Ions at the Cytoplasmic Interface

When the enzyme is kept in electrolyte without Na^+^ and K^+^ ions, the predominant conformation is affected by buffer substances present in the aqueous phase (Grell et al. 1991). In the presence of 30 mM imidazole, 1 mM EDTA and 5 mM Mg^2+^, the predominant conformation is E_1_. Adding increasing amounts of Na^+^ ions to the aqueous phase in the absence of ATP leads to successive occupation of the cation binding sites by Na^+^ until saturation is reached. During this titration experiments, the Na,K-ATPase remains entirely in the E_1_ conformation, and the partition equilibrium is distributed between the four states, E_1_ ⇄ NaE_1_ ⇄ Na_2_E_1_ ⇄ Na_3_E_1_, depending on the effective Na^+^ concentration. At physiological pH and in the absence of K^+^ the two ion-binding sites, which are able to bind K^+^, are, however, not empty but occupied by H^+^ so that binding of the first two Na^+^ is an exchange of H^+^ against Na^+^ (Apell and Diller 2002).

**Fig. 4:**
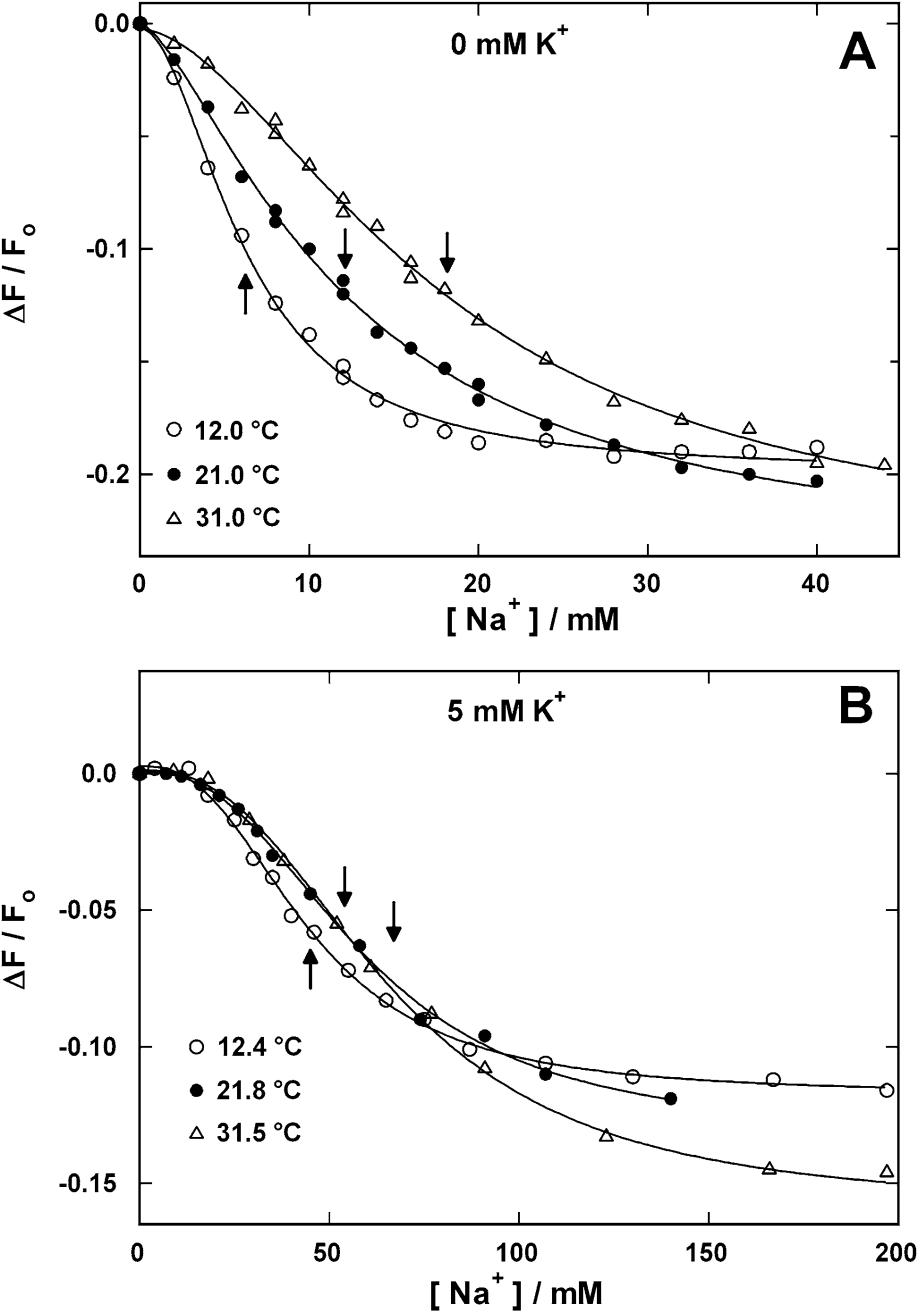
Na^+^ binding in the E_1_ conformation from the cytoplasmic side in the absence **(a)** and presence of 5 mM K^+^ **(b)**. In the presence of 30 mM imidazole, 1 mM EDTA, and 5 mM Mg^2+^, aliquots of a NaCl solution were added and the corresponding decrease steps of the RH421 fluorescence were recorded that are proportional to the occupation of the third Na^+^-binding site of the Na,K-ATPase (Schneeberger and Apell 1999). The concentration dependence was fitted by a Hill function and the temperature-dependent, apparent half-saturation concentration, *K*_1/2_, was determined (as indicated by arrows)

Titration experiments were performed with Na,K-ATPase-containing membrane fragments. The occupation of binding sites by Na^+^ ions was detected with RH421. The specific fluorescence *∆F*/*F*_o_ of the dye in the membrane declined with increasing occupation of the ion-binding sites. In Fig. 4a, Na^+^ titration experiments are shown for three different temperatures in the absence of K^+^. The concentration dependence could be fitted by a sigmoidal Hill function with a temperature-independent Hill coefficient, *n*_H_, of 1.7  ±  0.2. The apparent half-saturation concentration, *K*_1/2_, increased with temperature as indicated by arrows in Fig. 4a. When the temperature dependence of *K*_1/2_ was used to determine an activation energy with the Arrhenius equation, a value of *E*_A_ = 40.8  ±  6.1 kJ/mol was obtained. Whether the slight differences of the fluorescence levels at saturating Na^+^ concentrations were significant or not is not understood so far.

In an electrolyte containing 5 mM K^+^ and no Na^+^, the Na,K-ATPase is accumulated predominantly in the state E_2_(K_2_) when neither ATP nor inorganic phosphate are present. Addition of Na^+^ to the solution with increasing concentration shifts the binding equilibrium towards Na^+^ occupied states, until finally saturation is reached in state Na_3_E_1_ according to the reaction sequence proposed by the Post-Albers cycle, E_2_(K_2_) → K_2_E_1_ → … → Na_2_E_1_ → Na_3_E_1_. Such titration experiments were also performed at three different temperatures. The Na^+^-induced fluorescence decreases are presented in Fig. 4b. Under the chosen experimental condition, the sigmoidal shape of the concentration dependence could be fitted by a Hill function with a constant *n*_H_ of 2.6  ±  0.1 at all three temperatures. In contrast to the experiments without K^+^, saturation of the Na^+^ binding sites was found only at concentrations above 150 mM. This observation and the fact that a significantly weaker temperature dependence was detected indicated that a different reaction step controlled the adjustment of the steady state in the course of the titration. The saturating fluorescence decease was temperature dependent with significant differences at 200 mM Na^+^. An activation energy of *E*_A_ = 15.5  ±  2.3 kJ/mol was found for the temperature dependence of the half-saturation Na^+^ concentrations, K_1/2_, which was determined by fits to the data. The low value of *E*_A_ (which is typical for diffusion-controlled processes) as well as the enhanced Hill coefficient hint that the displacement of K^+^ by Na^+^ played a major role in this partial reaction.

### Transport-Related Reaction Steps

To determine the activation energy of single reaction steps of the pump cycle related to ion transport experimental conditions had to be found, in which all pumps in the solution are accumulated initially in one specific state and upon a trigger signal only a single step should be performed. Such an attempt is hardly feasible. Even if conditions can be defined in which only a narrowly limited sequence of reaction steps is executed, the choice is further restricted by the fact that in addition it is necessary to find an appropriate trigger to start the reaction sequence for all pumps simultaneously. Such a concept excludes by principle the isolated investigation of reaction steps that occur spontaneously. Actually, conditions had to be chosen in which the Na,K-ATPase is initially confined to only a few neighboring states, with one of them occupied predominantly. To initiate the course of the desired reaction sequence, different approaches are applicable. The first one is the rapid addition of a specific substrate. When time-resolved kinetics shall be investigated, the first choice is the use of caged compounds, if available, to produce a stepwise increase. In the case of the Na,K-ATPase, the respective key player was caged ATP. This inactive precursor of the energizing substrate is split by a short UV flash (< 1 µs) and thus produces an increase of the ATP concentration (with a time constant of a few ms). The relaxation of the protein into a new steady state or equilibrium after release of ATP can be detected and analyzed (Kaplan et al. 1978; Stürmer et al. 1989). Further methods are stopped-flow (Taniguchi et al. 1983; Kane et al. 1997) and quenched-flow techniques (Kane et al. 1997; Cornelius 1999) as well as voltage-jump or charge-pulse experiments (Nakao and Gadsby 1986; Bahinski et al. 1988; Wuddel and Apell 1995). When more than one reaction step is involved in the initiated process, it is important to ensure that the step of interest is made rate-limiting to allow the analysis of its specific kinetic properties with appropriate effort and precision.

**Fig. 5:**
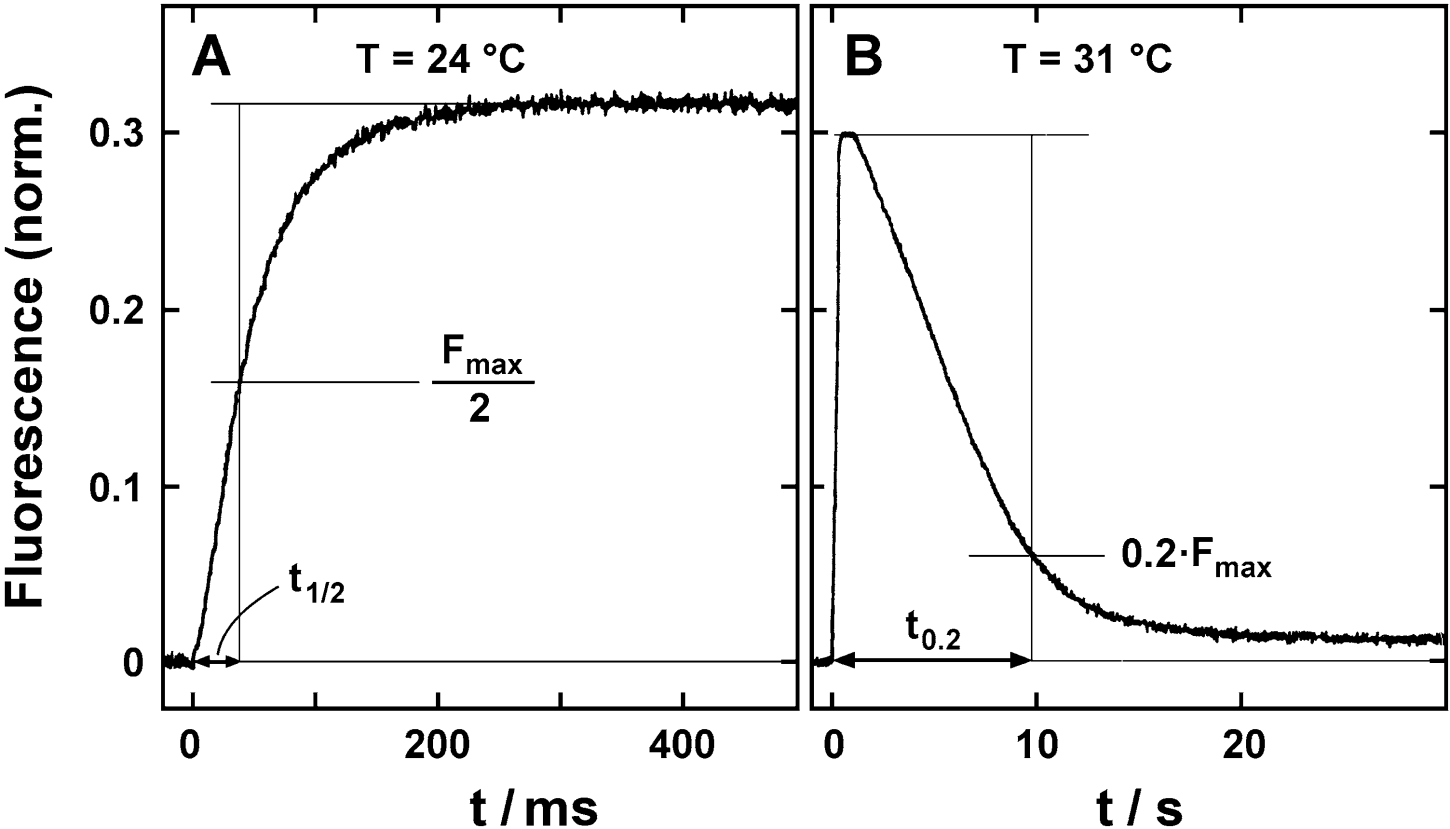
Response of the Na,K-ATPase upon an ATP concentration jump that started pump activity at *t* = 0. The enzyme was kept in buffer containing 100 mM NaCl so that the pumps were maintained in the Na_3_E_1_ state when ATP was released from caged ATP by a UV-light flash. The RH421 fluorescence responded on the release of the 3 Na^+^ after the transition to the P-E_2_ conformation of the pump. In panel **(a)**, ~ 20 µM ATP were released and the fluorescence level obtained after about 200 ms was constant for many seconds. The kinetics of the process was characterized by *t*_1/2_, the time when the fluorescence increase was half maximal. In panel **(b)**, the experiment was repeated with ~ 0.4 µM ATP released. This led to a rapid depletion of the released ATP, and after a few seconds, the supplementation of state E_2_P by enzyme phosphorylation ceased and the dominant partial reaction became the return reaction from the P-E_2_ state to Na_3_E_1_. This process was characterized by *t*_0.2_, the time when the fluorescence intensity decreased to the level of 20% of the maximum value

Two typical experiments, in which ATP-induced partial reactions were performed, are shown in Fig. 5. Purified Na,K-ATPase in membrane fragments were suspended in a fluorescence cuvette with 300 µl buffer containing 25 mM histidine, pH 7.2, 5 mM MgCl_2_, 1 mM EDTA, 100 mM NaCl, 100 µM caged ATP, and 200 nM RH421. At time *t* = 0, ATP was released by a UV flash from its inactive precursor. A fluorescence increase was detected that saturated after about 300 ms. This response represented the expected partial reaction that started from state Na_3_E_1_ which is virtually the only state formed in the presence of the chosen saturating (cytoplasmic) Na^+^ concentration. Upon the ATP concentration jump from 0 to ~ 20 µM, the reaction sequence, Na_3_E_1_ → Na_3_E_1_ATP → (Na_3_)E_1_-P → P-E_2_Na_3_ → P-E_2_, was triggered. Due to the significantly lower binding affinity for Na^+^ in the P-E_2_ conformation preferentially all three Na^+^ ions were released to the aqueous phase which caused the observed fluorescence increase (Heyse et al. 1994). A suitable parameter to quantify this relaxation process that is primarily controlled by the rate-limiting reaction step in the reaction sequence is the time, *t*_1/2_, in which the half-maximum fluorescence increase is achieved, as indicated in Fig. 5a. At 24 °C, *t*_1/2_ was 38  ±  2 ms. To derive the activation energy of this partial reaction, *t*_1/2_ was determined as function of temperature. Control experiments with 200 µM caged ATP resulted in the same *t*_1/2_ values. This meant that the time course of ATP release and binding at the used 100 µM did not affect the kinetic behavior.

To confine further the rate-limiting step in this induced reaction sequence, the experiments were repeated with 5-IAF-labeled enzyme. The covalently bound fluorescent dye responds to the conformation transition step, (Na_3_)E_1_-P → P-E_2_Na_3_ (Steinberg and Karlish 1989; Stürmer et al. 1989). The relative fluorescence changes were measured and analyzed in a similar way and exhibited characteristic time constants, *t*_1/2_, not significantly different from that of the RH421 signal (not shown) as reported earlier (Heyse et al. 1994). These findings supported the assumption that the conformation transition is the rate-limiting step in this partial reaction.

When the RH421 signal was recoded for a longer time period, a biphasic signal could be observed. In Fig. 5b an experiment is shown performed at 31 °C with a reduced initial concentration of 2 µM caged ATP. After a fast rise to a maximum, the fluorescence signal began to decline after about 500 ms due to a depletion of the released ATP in the solution. Under the chosen experimental conditions by the UV flash, about 4 ATP molecules were released per Na,K-ATPase. With a turnover rate of about 2 s^−1^ at 31 °C (as derived from Fig. 3), the supply of ATP was consumed within seconds. The initial rise of the fluorescence intensity was caused by release of the 3 Na^+^ after the transition to the P-E_2_ conformation (Fig. 5a). In the absence of K^+^, the state P-E_2_ was predominant after the conformation transition, since the transition back to the E_1_ was the slowest process in the reaction cycle. After depletion of ATP, further supplementation of state E_2_P ceased, and the slow partial reaction, P-E_2_ → E_2_ → E_1_ → Na_3_E_1_, took over. This process caused the decrease of the fluorescence intensity which reflected the increasing amount of ion pumps trapped in the Na^+^ bound state, Na_3_E_1_, which is characterized by the lowest RH421 fluorescence emission. The decline of fluorescence was parameterized by the time *t*_0.2_, when the amplitude had decreased to the level of 20% of the maximum value, as indicated in Fig. 5b.

**Fig. 6:**
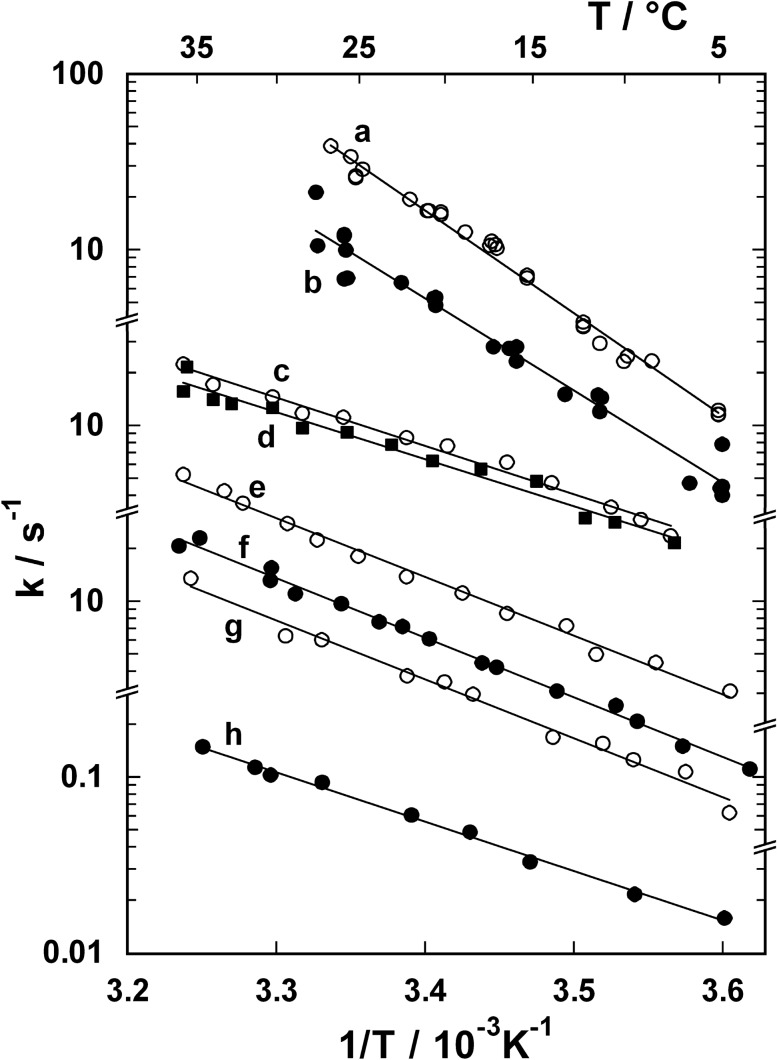
Arrhenius plots of various partial reactions of the Na,K-ATPase. The apparent rate constants of the investigated partial reactions were derived from the experimental values *t*_1/2_ or *t*_0.2_ (as introduced in Fig. 5) in the temperature range between 5 °C and 35 °C. The assignment of traces **a** to **h**, the corresponding experimental conditions, and the activation energies *E*_A,app_ are given in Table 1. The straight regression lines fitting the data indicate that a single reaction step was rate limiting in the covered temperature range

To evaluate activation energies of various steps of the pump cycle 8, different series of experiments were performed in a temperature range between 4 °C and 36 °C. Rate limitation of different reaction steps was obtained by appropriate choice of substrate concentrations. Apparent rate constants, *k*, were derived by the relation *k* = 1/t*, where t* was either *t*_1/2_ or *t*_0.2_ (as introduced in Fig. 5). The results are shown in Fig. 6. The temperature dependence of *k* is represented as Arrhenius plot and was used to calculate apparent activation energies, *E*_A,app_. To detect the respective pump activities, experiments were performed with two fluorescent probes, 5-IAF and RH421. Common to all experiments was the buffer composition of 25 mM histidine, pH 7.2, 5 mM MgCl_2_, and 1 mM EDTA. The essential experimental conditions, the determined activation energies, and the involved rate constants as well as the assumed rate-limiting steps are compiled in Table 1. Each series of measurements was repeated at least two times. The differences between corresponding experimental series, mostly performed with different enzyme preparations, were not significant. The resulting apparent activation energies in Table 1 are the average values ± standard error. For the sake of clarity, only a single set of experiments is presented for each condition in Fig. 6. As can be seen in Table 1, variation of the substrate concentrations of Na^+^, K^+^, and ATP allowed a modification of the predominant rate constant in the proceeding partial reactions. To justify this approach, two assumptions had to be made: In the course of the time-resolved kinetic experiments (1) backward directed rate constants (i.e. rate constants with the index “b” in Fig. 1) did not contribute significantly to the observed processes in the time period chosen for the measurement, and (2) the effect of the activation energy of ion binding to and release from ion sites could be neglected, since these actions were diffusion controlled and thus were significantly faster than all other participating reaction steps at each temperature applied in the experiments. In contrast to the Arrhenius plots derived from experiments investigating enzyme activity and ion transport under steady-state turnover conditions of the Na,K-ATPase (Figs. 2 and 3), the Arrhenius plots of all investigated partial reactions resulted in straight lines as can be seen from Fig. 6.

**Table 1:** Determination of apparent activation energies

trace (Fig. 6)	dye	[X^+^] /mM	[ATP] /µM	Involved kinetic constants (cf. Figure 1)	time constant /s (at 20 °C)	*E*_A,app_ /kJ/mol
a	RH421	150 Na^+^	20	*a*_f_, *p*_f_, ***l***_**f**_,$K_{{{\text{Na}}}}^{{{\text{ext}}}}$	0.071	111.7 ± 2.3
b	5-IAF	140 Na^+^ + 10 K^+^	20	*a*_f_, *p*_f_, ***l***_**f**_, $K_{{{\text{Na}}}}^{{{\text{ext}}}}$, $K_{{\text{K}}}^{{{\text{ext}}}}$, *q*_f_, *s*_f_, ***k***_**f**_	0.200	100.3 ± 4.0
c	RH421	150 Na^+^	0.1	***a***_**f**_, *p*_f_, *l*_f_,$K_{{{\text{Na}}}}^{{{\text{ext}}}}$	0.133	52.8 ± 1.9
d	5-IAF	150 Na^+^ + 2 K^+^	0.1	***a***_**f**_, *p*_f_, *l*_f_, $K_{{{\text{Na}}}}^{{{\text{ext}}}}$, $K_{{\text{K}}}^{{{\text{ext}}}}$, *q*_f_	0.159	51.3 ± 2.3
e	RH421	0.5 Na^+^	20	*a*_f_, ***p***_**f**_, *l*_f_,$K_{{{\text{Na}}}}^{{{\text{ext}}}}$	0.909	63.9 ± 1.7
f	5-IAF	1 K^+^	20	*s*_f_, ***k***_**f**_	0.167	64.9 ± 1.3
g	5-IAF	1 K^+^	0.8	***s***_**f**_, *k*_f_	0.270	64.3 ± 2.5
h	RH421	150 Na^+^	0.2 → 0	*a*_f_, *p*_f_, *l*_f_, $K_{{{\text{Na}}}}^{{{\text{ext}}}}$, ***r***_**f**_	16.667	53.7 ± 1.3

Experimental conditions and apparent activation energies, *E*_A,app_, for different partial reactions. Temperature dependence of the corresponding characteristic rate constants is shown in Fig. 6. The proposed rate-limiting kinetic constants are given in bold in the list of rate constants participating in the respective partial reactions (column 5). The time constants at 20 °C were calculated as 1/*k*, whereby the corresponding values of *k* were taken from the regression lines in Fig. 6

The activation energies noted in Table 1 are apparent quantities, since they are derived from a series of consecutive reaction steps that constitute the triggered partial reaction of the pump cycle. The fact that the data points followed a straight line indicated, however, that only a single rate-limiting step (or potentially, more than one step with comparable activation energies) controlled the reaction kinetics that was represented by the observed time constant in the covered range of temperatures.

In the Na-only mode of the pump (i.e. 0 K^+^), experiments were performed in the presence of 150 mM NaCl and saturating ATP concentrations. From earlier experiments it is known that under these conditions the return reaction, P-E_2_ → E_1_, is the slowest of the pump cycle in the order of seconds (Fig. 5b) and may be neglected in time-resolved experiments that last only 400 ms (Heyse et al. 1994). During this period the conformation transition, (Na_3_)E_1_-P → P-E_2_(Na_3_), with the rate constant *l*_f_ is the limiting step (Stürmer et al. 1989). The apparent activation energy of 112 kJ/mol was the highest value observed for single reaction steps in this study (Fig. 6, trace a). When the experiments were repeated with 5-IAF labeled enzyme and in the presence of 140 mM Na^+^  + 10 mM K^+^, the Na,K mode was activated, and the rate-limiting step of the second half of the pump cycle contributed additionally to the kinetic behavior. At ATP concentrations > 2 µM binding of ATP to the low-affinity binding site caused an accelerated return to the E_1_ configuration, E_2_(K_2_) → ATP‧E_2_(K_2_) → K_2_E_1_‧ATP. This partial reaction is fast when compared to the reaction step ensuing after enzyme dephosphorylation at concentrations significantly below 2 µM ATP, E_2_(K_2_) → K_2_E_1_. Starting from state Na_3_E_1_, upon release of 20 µM ATP the system relaxed mostly into a mixture of the states (Na_3_)E_1_-P and ATPE_2_(K_2_). They are the two states preceding the rate-limiting steps in the Na^+^ and K^+^-transporting half cycles of the pump (cf. Figure 1), which were assigned to be controlled by *l*_f_ and *k*_f_, respectively (Heyse et al. 1994). In principle, the reaction E_2_(K_2_) → K_2_E_1_ with the rate constant *r*_f_ is also possible, however, it may be neglected in these experiments, since the time constant of this conformation transition, 1/*r*_f_, is large (> 2 s) compared to observed value of 1/*l*_f_ (50 ms at 20 °C) and the duration of the recorded experiments (400 ms). In this set of experiments a lower activation energy of 100 kJ/mol was determined (Fig. 6, trace b, and Table 1).

At a concentration of 100 nM released ATP, below the high affinity dissociation constant (~ 200 nM), ATP binding became rate-limiting, the predominant reaction may be expected to be Na_3_E_1_ → Na_3_E_1_ATP, and the corresponding rate constant is *a*_f_ (Fig. 1). These experiments were performed in 150 mM NaCl using RH421 and in 150 mM NaCl + 2 mM KCl with 5-IAF labeled enzyme. Within the duration of the recorded fluorescence signal (400 ms), the total reaction sequence could be assumed to be restricted to Na_3_E_1_ → … → P-E_2_ in the absence of K^+^, and to Na_3_E_1_ → … → E_2_K_2_ in the presence of 2 mM K^+^. For both experimental conditions the same apparent activation energy of 52 ± 2 kJ/mol was determined (Fig. 6, traces c and d, and Table 1).

At low sodium concentration (0.5 mM) before the release of ATP a steady state was obtained, in which the state Na_3_E_1_ was populated only to minor extent (cf. Figure 4a). Upon release of high ATP (20 μM) at time 0, according to the site occupation in the current steady-state distribution, E_1_ ⇄ NaE_1_ ⇄ Na_2_E_1_ ⇄ Na_3_E_1_, binding of one or more Na^+^ ions from the aqueous phase has to occur to reach the state Na_3_E_1_ which eventually is phosphorylated and triggers the partial reaction detected by RH421. Therefore, Na^+^ binding becomes rate-limiting and contributes significantly to the time course of the RH421 fluorescence increase and the observed temperature dependence of *t*_1/2_. In Fig. 6, trace e, corresponding data are shown from which an activation energy of 64 kJ/mol was calculated that was significantly lower than that determined at high Na^+^ (trace a). According to a previously published study the rate-limiting step in this partial reaction was proposed to be a conformational rearrangement of the pump preceding binding of the third Na^+^ (Schneeberger and Apell 1999).

In the presence of K^+^ and absence of Na^+^ the Na,K-ATPase adopted a steady-state distribution between both states, K_2_E_1_ ⇄ E_2_(K_2_). In buffer with saturating 1 mM K^+^ the enzyme is trapped quantitatively in the occluded state, E_2_(K_2_). Upon release of ATP the reaction sequence E_2_(K_2_) → ATPE_2_(K_2_) → K_2_E_1_ATP was triggered. At 0.8 µM ATP, the first reaction, binding of the nucleotide to the low-affinity site (K_M_ ≈ 2 μM), had a significant effect on the rate-limiting step, governed by *s*_f_ (Fig. 1). In contrast, when 20 µM ATP ($\gg$ *K*_M_) were released the subsequent conformation transition was expected to be limiting with the rate constant *k*_f_. In both conditions 5-IAF labeled enzyme was used to detect the partial reaction triggered by an ATP concentration jump. The corresponding activation energies have been found to be comparable, 64 kJ/mol (Table 1) at high ATP (Fig. 6, trace f) and low ATP (Fig. 6, trace g).

In a last series of experiments the reaction P-E_2_ → E_1_ was investigated in the Na-only mode. As shown in Fig. 5b, when pump function was started at time 0 by release of a limited pulse of ATP (0.2 µM at *t* = 0), the enzyme proceeded initially from Na_3_E_1_ to state P-E_2_ and was preferentially accumulated there, because in the absence of K^+^ the return to the E_1_ conformation with the rate constant *r*_f_ (Fig. 1) is slow. When the pool of ATP wore out, enzyme that had returned to state Na_3_E_1_ was no longer rephosphorylated but remained trapped with 3 Na^+^ bound, which is the state with the lowest RH421 fluorescence level (Fig. 5b). Experimental conditions were chosen so that in the cuvette less than 4 ATP molecules per pump are released by the light flash. With a pump rate of approximately 2 s^−1^ at 31 °C (according to Fig. 3) ATP-facilitated turnovers ceased after a few seconds (condition of Fig. 5b). At longer times (*t* > 5 s) the observed fluorescence decrease was controlled by the rate-limiting reaction step of the partial reaction from P-E_2_ → … → Na_3_E_1_. The kinetic behavior was parametrized by the time constant *t*_0.2_ (Fig. 5b) and used to derive the associated activation energy. The apparent activation energy obtained from this set of experiments was found to be 54 kJ/mol (Fig. 6, trace h).

When the activation energies obtained from the bent course of the pump turnover experiments in the Arrhenius plots (Figs. 2 and 3) were compared with the values derived from ‘single’ reaction steps, it was not so easy to assign those to the processes that control the turnover in the low and high temperature range. One reason was that part of the experiments was performed with reconstituted vesicles and hence the enzyme molecules were embedded in an artificial lipid environment. It was shown earlier that the composition of the lipid matrix has significant effects on the activation energy of the enzyme activity (90 kJ/mol < E_A_ < 160 kJ/mol) and that suboptimal compositions lead to an increase of the observed activation energies (Marcus et al. 1986). The series of experiments in which the enzyme activity was determined in membrane fragments revealed values of *E*_A, low T_ = 118 kJ/mol and *E*_A, high T_ ~ 46 kJ/mol. Under turnover condition the apparent values of *E*_A_ have to be expected to be a somehow weighted blend and interaction of all participating reaction steps. Since the substrate concentrations in the turnover experiments were chosen to be not limiting, the contribution of substrate-dependent steps should be, however, less significant. Therefore, it appears that the rate-limiting conformational changes at the lower temperature more closely approximates that of (Na_3_)E_1_-P → P-E_2_Na_3_ (*E*_A_ = 112 kJ/mol) and at higher temperatures that of ATP‧E_2_(K_2_) → K_2_E_1_‧ATP (*E*_A_ = 65 kJ/mol), respectively.

## Discussion

The role of active ion transporters in living cells is evident. Due to the second law of thermodynamics processes can occur only in the direction in which the overall entropy increases. The unavoidable consequence of this principle for living cells would be that all ion concentrations gradients and the electric potential across membranes will break down with time and end up in a perfect equilibrium between all compartments of a cell and its environment. To prevent such a lethal development specific counteracting transporters exist in membranes, which separate the various compartments. They are called active transporters and utilize energy from various sources to transfer vital substrates across membranes to build up and maintain electrochemical potential gradients. Due to thermodynamical principles, however, only part of the energy involved in this process, the so-called Gibbs free energy, *G*, can be used for the desired transport, the remainder gets lost irreversibly in form of entropy by heat production. The yield of beneficial energy depends on the properties of the molecular process.

In the case of so-called primary active ion transporters such as ion-transporting ATPases the conversion efficiency, *η*, of the overall performance can be easily determined by relating the free energy stored by the transport process, Δ*G*_transp_, as electrochemical potential gradient across the membrane and the amount of free energy provided by ATP hydrolysis, Δ*G*_ATP_. It is calculated as *η* = Δ*G*_transp_/Δ*G*_ATP_. Depending on the substrate conditions efficiencies up to 90% may be found (Läuger 1991).

In case of the F_0_F_1_ ATPase the molecular mechanism of energy conversion from ATP hydrolysis to H^+^ transport was analyzed in meticulous manner and resulted in the discovery of an impressively effectively interacting aggregate of more than twenty protein subunits that operates as proton translocator with a stator and rotor (Junge et al. 2001; Junge and Nelson 2015). The pathway of the translocated protons as well as the movements of the relevant protein domains facilitated by ATP binding, hydrolysis and ADP release are now understood on a molecular level. Proton pumping by the light-driven bacteriorhodopsin was also clarified with high precision on a molecular level including the movements of single amino acid sidechains and even functional water molecules (Lanyi and Schobert 2003; Wickstrand et al. 2015). In contrast, in case of P-type ATPases mechanistic concepts are still missing of how the chemical energy provided by ATP hydrolysis is transformed into an electrochemical potential gradient. While the proposed pathways for the transported ions are rather convincing for the SR Ca-ATPase (Møller et al. 2010), Na,K-ATPase (Apell 2019), and the KdpFABC complex (Pedersen et al. 2019), mainly based on structural investigations with high resolution, a mechanistic concept of energy transduction, however, could not be advanced beyond speculation so far.

The experimental results available at present and some basic thermodynamic principles may be used, however, to define constraints that have to be met when concepts are proposed to explain the molecular mechanism of energy transduction in P-type ATPases, especially in the case of the Na,K-ATPase.

One general constraint was deduced by Terell L. Hill who showed that energy transduction in molecular machines does not occur in a single step of the reaction cycle (here: the pump cycle of the Na,K-ATPase) but is distributed over the whole cycle (Hill 1977, 1989). This finding contradicts the principle of a “power stroke” as it is known from macroscopic engines. To verify this proposition in case of the Na,K-ATPase it has to be determined to what extent each single reaction step of the pump cycle contributes to storage and consumption of the system’s free energy in terms of changes of the so-called “basic free energy levels”, Δ*µ*_i,j_ (Läuger 1991; Apell 1997). These quantities may be calculated from the forward and backward rate constants, *k*_i_ and *k*_j_, respectively, (or the equilibrium constant *K*_i,j_) of the transition between two neighboring states, *i* and *j*, of the pump cycle as.

$$\Delta \mu_{i,j} \,\, = \,\,RT \cdot \ln \left( {k_{j} /k_{i} } \right)\;\; = \;\; - RT \cdot \ln \left( {K_{i,j} } \right)$$

where *R* is the gas constant and *T* the absolute temperature. In this notation *k*_i_ and *k*_j_ may be substrate-dependent quantities. Positive values of Δ*µ*_i,j_ (when advancing from state *i* to *j*) represent energy storage, negative values indicate energy dissipation, and values around 0 result from processes close to an equilibrium condition. Based on numerous experiments, in which the reaction kinetics of the Na,K-ATPase was investigated, the basic free energies of each reaction step in the pump cycle were determined. By compilation of the resulting values it could be shown that there was indeed no “power stroke” reaction step (Apell 1997).

**Fig. 7:**
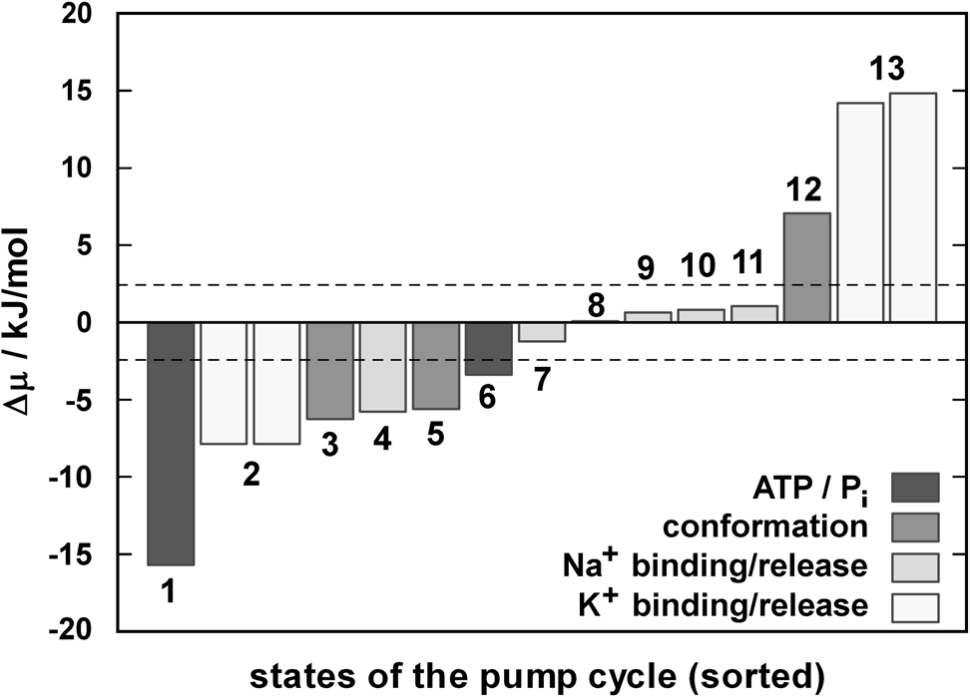
Changes of the basic free energy, Δµ, of single reaction steps forming the Post-Albers cycle of the Na,K-ATPase (Fig. 1) under physiological substrate concentrations. The steps are sorted according to their magnitude. Negative values indicate energy dissipation, positive values energy consumption, and values around 0 reactions close to equilibrium. The dashed lines represent the thermal energy at room temperature. ATP binding (1) is the most prominent energy-dissipating reaction step. But also the substrate-independent steps enzyme phosphorylation together with Na^+^ occlusion (3), conformation transition, E_1_-P → P-E_2_ (5), and dephosphorylation together with K^+^ occlusion (6) are energy-dissipating steps, while the conformation transition back to E_1_ (12) was an energy consuming step related to a conformational rearrangement. The significant energy dissipation when binding both K^+^ (2) and energy consumption when of both K^+^ are released to the cytoplasm (13) is a consequence of ion concentrations far off the respective dissociation constants. While release of the second Na^+^ to the extracellular side (4) is a dissipating step, binding or release of the other Na^+^ (7 – 11) were processes close to the thermodynamic equilibrium (Δµ < RT)

When experimentally determined rate constants of the Post-Albers cycle (Fig. 1) were used under (near) physiological conditions, i.e.[Na^+^]_cyt_ = 5 mM, [K^+^]_cyt_ = 150 mM, [Na^+^]_ext_ = 140 mM, [K^+^]_ext_ = 5 mM, [Mg^2+^]_cyt_ = 5 mM, [ATP] = 5 mM, [ADP]_cyt_ = 0.1 mM, [P_i_]_cyt_ = 5 mM, T = 20 °C, and membrane voltage, *V*_m_ = 0, the respective Δ*µ*_i,j_ values could be calculated from published rate constants (Apell 1997). They are shown in Fig. 7, ordered in ascending sequence. It was found that the largest decrease of free energy occurred at low-affinity ATP binding, E_2_(K_2_) + ATP → ATP‧E_2_(K_2_), with a value of −15.7 kJ/mol. Significant changes of basic free energy were observed also in extracellular K^+^ binding (−7.8 kJ/mol) and cytoplasmic release (+ 14.5 kJ/mol) of K^+^. Those values far away from the thermodynamic equilibrium provoke a strong shift in the respective reaction steps to the product state (if Δ*µ* < 0) or initial state (if Δ*µ* > 0). Conformational rearrangement such as enzyme phosphorylation together with Na^+^ occlusion, Na_3_E_1_‧ATP → (Na_3_)E_1_-P, and enzyme dephosphorylation together with K^+^ occlusion, P-E_2_K_2_ → E_2_(K_2_) + P_i_, or the major conformation transitions, (Na_3_)E_1_-P → P-E_2_Na_3_ and ATP‧E_2_(K_2_) → K_2_E_1_‧ATP were characterized by |Δ*µ*|< 7 kJ/mol. Except for the release of the second Na^+^ on the extracellular side (Δ*µ* ~ 6 kJ/mol), a step which includes a significant conformational rearrangement (Wuddel and Apell 1995; Holmgren et al. 2000), the other Na^+^ binding and release reactions exhibited |Δ*µ*| values < 2 kJ/mol, which are smaller than the thermal energy at 20 °C (RT = 2.43 kJ/mol, dashed lines in Fig. 7) and close to thermodynamical equilibrium.

The large decrease of basic free energy in the case of ATP binding has to be attributed to the fact that the ATP concentration of 5 mM used in the calculation is large compared to the equilibrium dissociation constant, *K*_ATP_, of ~ 10 µM (Schuurmans Stekhoven and Bonting 1981; Läuger 1991), which affects significantly the value of Δ*µ*. The same argument holds for magnitude of Δ*µ* of K^+^ binding and release with equilibrium dissociation constants *K*_K,ext_ of ~ 0.2 mM and *K*_K,cyt_ of ~ 2.5 mM, respectively. The values of Δ*µ* of the substrate-dependent reaction steps may be varied significantly without considerable effect on the pump activity. When for example the ATP concentration of 5 mM is reduced to 150 µM, which is still large compared to *K*_ATP_ of 10 µM, the decrease of the basic free energy changes of −15.7 kJ/mol (Fig. 7) is cut in half without detectable effect on the experimentally obtained pump activity.

According to Fig. 7 cytoplasmic K^+^ release is the process in which under physiological conditions the highest amount of energy (~ 14 kJ/mol per ion) is “transferred” from the pump to the cell. The fact that K^+^ release occurs at all under this condition is due to several properties of the ion pump and the cell: (1) binding and release of ions are diffusion-controlled processes, i.e. ion exchange occurs within a µs time frame. Therefore, a steady-state distribution between all states is obtained persistently under physiological conditions for the respective reaction sequence, K_2_E_1_⋅ATP ↔ KE_1_⋅ATP ↔ E_1_⋅ATP ↔ NaE_1_⋅ATP ↔ Na_2_E_1_⋅ATP, because the preceding and subsequent reaction steps, which are conformation transitions, are slow compared to ion exchange. At physiological ion concentrations in the order of 100 mM K^+^ and 5 mM Na^+^, only a very small fraction of pumps is in state Na_2_E_1_⋅ATP. (2) Binding of the third Na ^+^ occurs only upon a minor conformational rearrangement of the pump, Na_2_E_1_⋅ATP → Na_2_E^*^_1_⋅ATP, that provides access to the third, Na^+^-specific binding site (Schneeberger and Apell 1999), and this process is slow compared to the ion-binding and release steps (Apell et al. 2017). This transition increases the probability that the Na_3_E_1_⋅ATP state is long-living enough to be drained by autophosphorylation of the Na,K-ATPase upon binding of a third Na^+^, Na_2_E^*^_1_⋅ATP + Na^+^  ↔ Na_3_E^*^_1_⋅ATP → (Na_3_)E_1_-P + ADP. (3) The transition to the Na^+^-occluded state is correlated with the transfer of P_i_ from the nucleotide to the enzyme. This process is a step with a significant decrease of the basic free energy of the ion pump (−6.2 kJ/mol) and therefore biased into forward direction. (4) The subsequent major conformation transition, (Na_3_)E_1_-P → P-E_2_Na_3_, exhibits also a significant decrease of the basic free energy (−5.7 kJ/mol) and thus amplifies the progress forward through the pump cycle. (5) Since the diffusion-controlled ion exchange in the non-occluded states of E_1_ is fast compared to the subsequent conformational rearrangements and enzyme phosphorylation, a low (but constant) population of the Na_2_E_1_⋅ATP is provided that is drained in forward direction and allows a continuous flux through the pump cycle. (6) The low population of the Na_2_E_1_⋅ATP state leads to the well-known effect that the turnover rate is under physiological K^+^ concentration significantly lower than at low concentrations or in the absence of K^+^ in the cytoplasmic aqueous phase (Skou 1975).

Another constraint is provided by the observation that upon substrate-related reaction steps always a conformational rearrangement (or more appropriate: a conformational relaxation) occurs as was reported for various steps in the pump cycle: (1) In the E_1_ conformation after binding of the second Na^+^ and before binding of the third Na^+^, Na_2_E_1_⋅ATP → Na_2_E^*^_1_⋅ATP (Schneeberger and Apell 2001), (2) after binding of the third Na^+^, Na_3_E^*^_1_⋅ATP → Na_3_E^#^_1_⋅ATP, thus enabling enzyme phosphorylation (Schneeberger and Apell 1999), (3) upon enzyme phosphorylation by ATP provoking ion occlusion, Na_3_E^#^_1_⋅ATP → (Na_3_)E_1_-P (Glynn and Karlish 1990), (4) after the conformation transition into the P-E_2_ conformation the first deoccluded Na^+^ ion is released with high electrogenicity, before a conformational relaxation leads to the release of the remaining two Na^+^ with low electrogenicity through a wide access channel, P-E_2_Na_2_ → P-E^*^_2_Na_2_ (Wuddel and Apell 1995; Holmgren et al. 2000), (4) when in P-E_2_ two K^+^ are bound enzyme dephosphorylation occurs associated with ion occlusion, P-E_2_K_2_ → E_2_(K_2_) (Forbush 1987; Forbush 1988), and (5) upon low-affinity binding of ATP in the E_2_ state the conformation transition, ATP⋅E_2_(K_2_) → K_2_E_1_⋅ATP, is significantly accelerated (Karlish 1979).

Based on these constraints a possible proposal to gain access to the molecular mechanism of the Na,K-ATPase could be that the transfer of energy-rich phosphate from ATP to the enzyme generates a protein conformation in which the energy is stored in a specific structural arrangement. Enzyme phosphorylation is not accompanied by a large change of the basic free energy (Fig. 7) that can be related to the ~ −55 kJ/mol provided by ATP hydrolysis. This fact may be explained by knowledge obtained from the F_0_F_1_ ATPase. There it was shown that the transfer of P_i_ between ADP and protein occurs close to the thermodynamic equilibrium due to a specific spatial environment in which this process takes place (Boyer 1989). In the case of the ATPase mode of this protein (which works in reverse mode as ATP synthase in mitochondria) the energy provided by ATP is transferred upon binding to the protein structure by a conformational rearrangement in which the shell of water molecules is displaced from ATP and the bare nucleotide fits perfectly into its binding pocket formed by the protein.

In case of the Na,K-ATPase it may be proposed that the energy provided by transfer of the phosphate from ATP to the protein is distributed rapidly over the protein by rearrangements of amino acid side chains in response to the coordination of the high-energy phosphate in the nucleotide binding pocket, thus creating (minor) changes in spatial alignments, mechanical tension and torque of protein helices, as well as modified electrostatic interaction between amino acid side chains and specific dipole movements. This way enhanced potential energy is transiently buffered in various subdomains of the protein structure. The first ‘response’ of the protein upon phosphorylation would be the conformation transition to P-E_2_. Subsequently, a meticulously concerted sequence of relaxation processes passes off such as deocclusion of the first Na^+^, followed by its release, which in turn induces a rearrangements of transmembrane helices that form the widened access channel between ion-binding sites and the aqueous phase and so unlock and trigger the release of the remaining 2 Na^+^, accompanied by a spatial adaption of the ion sites to a shape that prefers K^+^ binding. Furthermore, the occupation of both binding sites by K^+^ that are now optimally coordinated allows a structural relaxation that causes their occlusion and release of the “low-energy” phosphate. The alternation of substrate binding or release steps and further structural relaxations (or rearrangements) continues until the circle is completed and the enzyme has arrived again in the state in which it is enabled to be phosphorylated and energized anew.

This is just a model for further discussion. Additional structural details of closely neighboring states of the pump cycle with atomic resolution may produce confirming or modifying insights. Perhaps this concept will trigger inspiring ideas for additional kinetical experiments that provide new crucial insights into the molecular mechanism of active ion transport by the Na,K-ATPase.
